# A comparison of baleen whale density estimates derived from overlapping satellite imagery and a shipborne survey

**DOI:** 10.1038/s41598-020-69887-y

**Published:** 2020-07-31

**Authors:** C. C. G. Bamford, N. Kelly, L. Dalla Rosa, D. E. Cade, P. T. Fretwell, P. N. Trathan, H. C. Cubaynes, A. F. C. Mesquita, L. Gerrish, A. S. Friedlaender, J. A. Jackson

**Affiliations:** 1 British Antarctic Survey, High Cross, Madingley Road, Cambridge, CB3 0ET UK; 2 University of Southampton, University Road, Southampton, SO17 1BJ UK; 3 Australian Antarctic Division, Department of Agriculture, Water and the Environment, Australian Government, Channel Highway, Kingston, 7050 Australia; 4 Laboratório de Ecologia e Conservação da Megafauna Marinha, Instituto de Oceanografia, Universidade Federal do Rio Grande-FURG, Av. Itália km.8, Rio Grande, RS 96203-900 Brazil; 5 Hopkins Marine Station, Stanford University, 120 Ocean View Blvd, Pacific Grove, CA 93950 USA; 6 Institute for Marine Sciences, University of California Santa Cruz, 115 McAllister Way, Santa Cruz, CA 95006 USA

**Keywords:** Marine mammals, Ecology

## Abstract

As whales recover from commercial exploitation, they are increasing in abundance in habitats that they have been absent from for decades. However, studying the recovery and habitat use patterns of whales, particularly in remote and inaccessible regions, frequently poses logistical and economic challenges. Here we trial a new approach for measuring whale density in a remote area, using Very-High-Resolution WorldView-3 satellite imagery. This approach has capacity to provide sightings data to complement and assist traditional sightings surveys. We compare at-sea whale density estimates to estimates derived from satellite imagery collected at a similar time, and use suction-cup archival logger data to make an adjustment for surface availability. We demonstrate that satellite imagery can provide useful data on whale occurrence and density. Densities, when unadjusted for surface availability are shown to be considerably lower than those estimated by the ship survey. However, adjusted for surface availability and weather conditions (0.13 whales per km^2^, CV = 0.38), they fall within an order of magnitude of those derived by traditional line-transect estimates (0.33 whales per km^2^, CV = 0.09). Satellite surveys represent an exciting development for high-resolution image-based cetacean observation at sea, particularly in inaccessible regions, presenting opportunities for ongoing and future research.

## Introduction

Gathering data on cetacean distribution and densities has traditionally employed visual observers operating from various platforms, typically either ships, aircraft or land^1–5^. Much of our understanding about baleen whale population recovery and ecology depends on these methods^6–8^. In oceanic regions close to population centres, these methods are often used to monitor regional population densities^8–10^. However, regular applications of these methods are often constrained in remote, inaccessible regions, where their use represents a significant logistical and financial commitment^11^. Consequently, such surveys are infrequent, making monitoring of population trends more challenging.

In the Southern Ocean, the only comprehensive surveys south of 60° S (i.e. the putative summer foraging area for a range of cetacean species) were those undertaken by the International Whaling Commission (IWC) during the International Decade of Cetacean Research and the Southern Ocean Whale Ecosystem Research (IDCR SOWER) surveys, between 1978/9 and 2003/4. These surveys circumnavigated the continent three times, and based on these data Southern Ocean baleen whale recovery trends have been estimated^6,12,13^. However, small-scale, sometimes ad hoc studies are far more common. These are generally biased towards the most accessible regions of the Southern Ocean^14^, the Western Antarctic Peninsula^3,4,15–18^, with more limited studies also conducted in the Scotia Arc^19^, Weddell Sea^20^ and limited areas of East Antarctica^21,22^. The Southern Ocean represents a region, amongst others globally^11^, that would benefit from further research into the structure and functioning of the whole ecosystem^23^.

Recent developments in the use of Unmanned Aerial Systems (UAS) for surveys suggest that it may be possible to monitor and detect marine mammals remotely^24–26^, without the limiting factors associated with crewed flights^26^. Collection of aerial imagery also minimises the effects of animal movement (attraction/avoidance)^27^, associated with traditional ship-based survey platforms^1^. Additionally, sighting data collected on traditional in situ observer-based platforms can be impacted by observer fatigue^26^, whereas by generating a permanent record of a sighting, an image-based survey allows the analyst to revisit data. Image-based surveys therefore reduce the reliance of the data analyst on near-instantaneous observer decisions, a key source of perception bias for traditional distance sampling surveys. However, the cost of long-range UAS platforms and surveys currently far exceeds that of comparable aerial platforms, both for deployment^25^, and subsequent analysis^28^. An alternative approach for collecting overhead imagery and detecting whales at sea has been provided by the development of sub-metre or Very-High-Resolution (VHR) satellite imagery. Since the early 2000′s, applications of VHR imagery have been mainly developed for terrestrial species and environments, with only a few studies focussing on large marine species at sea^29–32^. Terrestrially focussed VHR studies have proven valuable for study species in remote regions, particularly the high latitudes^33–40^, where contrast between the target species and their visually-homogeneous environment makes their detection easier^41^. Comparisons between traditional methods and image-based techniques have only been undertaken in a few cases, notably in the high latitudes, where Emperor penguins *Aptenodytes forsteri*^34^, elephant seals *Mirounga leonina*^40^, wandering *Diomedea exulans* and royal albatross *D. sanfordi*^33^, and polar bears *Ursus maritimus*^36,42^ have been examined. All of these studies have demonstrated that VHR counts were comparable to traditional ground-based counts, highlighting the merit of this novel platform for remote ecological observations. However, to date, ground-to-space comparisons have been exclusively terrestrial, and there have been no attempts to compare animal densities estimated from traditional at-sea surveys to those obtained through VHR image analysis.

Applying VHR imagery for conservation research is in its infancy. However, the ability to rapidly and repeatedly task a satellite to acquire images from anywhere on the planet is a distinct advantage in ecological research, particularly given the resampling limitations facing traditional survey methods in remote regions stemming from the logistics and associated cost of such surveys. The emergence of this technique offers the possibility to hindcast analyses back to ca.2009, and the launch of the WorldView-2 satellite (when the resolution arguably became fine enough to discriminate features at sea^30^), and examine densities over the past decade in regions where imagery is available. The present study aims to provide the first assessment of space-borne, VHR image-derived density estimates for whales using this area, compared to those obtained from a traditional ship line-transect survey, in a region where traditional surveys are often constrained. We selected a remote region where species composition is dominated by a single species, and local geomorphology provides sheltered, low sea-state surface conditions well suited to satellite imagery analysis^30^. The region is the Gerlache Strait, Western Antarctic Peninsula (WAP), 63° 45′ S to 65° 00′ S, an area known to be a significant summer feeding area for cetaceans; notably humpback whales *Megaptera novaeangliae*^15,43^, where they typically comprise > 80% of sightings^15,16^.

## Results

### Ship survey

**Figure 1:**
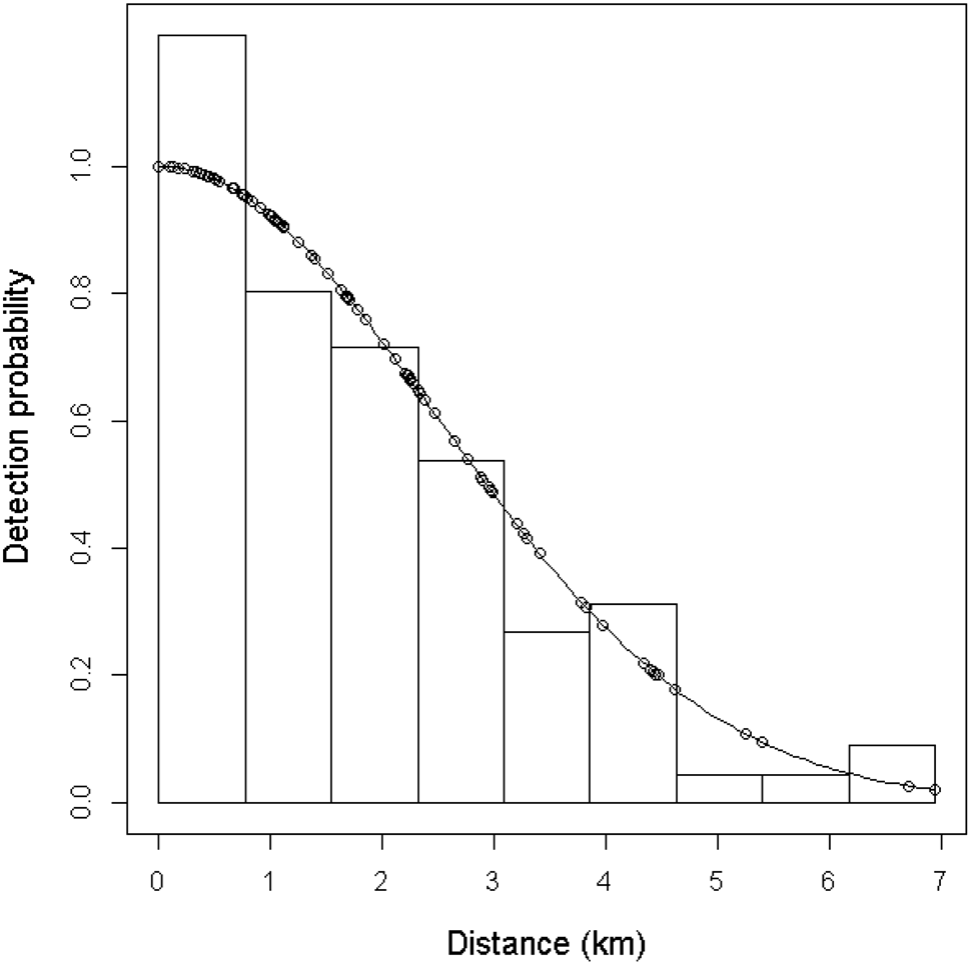
Half-normal model detection function with no adjustments and no covariates fitted to the perpendicular detection distances from the ship-borne line transect survey data.

Total line effort considered for this study was 90.7 km, with an effective half strip width estimated to be 3.1 km (CV = 0.07). A total of 90 groups (185 individuals; 95.7% humpback, n = 177, 85 groups; 2.7% unidentified large whale, n = 5, 4 groups; 1.6% fin, n = 3, 1 group) were encountered. Average group size was estimated as 2.06 individuals (CV = 0.01). Right truncation at 5% of the maximum perpendicular detection distance was tested but did not improve the fit of the models, so was not implemented. A half-normal key with no covariates best fit the data (CvM p = 0.376) (Fig. 1), but multiple models were within 2 AIC units of each other (Table 1). Parameter estimates were checked to rule out implausible models, and the most parsimonious model was selected. Along-track density was estimated at 0.33 whales per km^2^ (95%, CV 0.09).

**Table 1:** Summary outputs of the fitted detection function models.

Model	Key	Formula	CvM p-value	$\hat{P}_{a}$	*se* $\hat{P}_{a}$	$\Delta$AIC
1	half-normal	~ 1	0.376	0.447	0.031	0.000
5	half-normal	~ sightability	0.403	0.443	0.033	0.759
6	hazard-rate	~ 1	0.816	0.398	0.054	0.783
7	hazard-rate	~ Beaufort	0.949	0.317	0.061	1.002
2	half-normal	~ Beaufort	0.332	0.444	0.032	1.175
3	half-normal	~ visibility	0.537	0.434	0.034	2.053
10	hazard-rate	~ sightability	0.912	0.394	0.055	2.468
8	hazard-rate	~ visibility	0.957	0.071	0.086	3.161
4	half-normal	~ Beaufort + visibility	0.530	0.433	0.035	3.717
9	hazard-rate	~ Beaufort + visibility	0.963	0.078	0.091	3.867

Headings shortened represent: CvM, Crammer-von Mises test p-values; ${\hat{\text{P}}}_{{\text{a}}}$, the average detectability; and se $\widehat{ P}_{a}$, the standard error of $\hat{P}_{a}$. Models listed in order of AIC.

### VHR image analysis

Following manual scanning and classification of the images by the main observer (O1), a total of 18 “definite”, 21 “probable” and 146 “unclassified” features of interest (FOIs) were identified. A subset (n = 37, 20%) of these identified features was passed to three independent reviewers (R1–R3) for classification. Their scores were then compared to those of O1. The deviation between the FOIs scored by O1 and the average of the R1, R2, and R3 was less than 1 (mean = − 0.31, median − 0.33), and the associated variance of these averaged reclassifications spanned both above and below O1 classifications (Fig. S1), suggesting close concordance overall. No adjustment was therefore made to the FOI scores for O1 overall. The proportion of whales classified as “definite” and “probable” by O1 was 0.27, which was identical to the averaged proportion obtained by reviewers R1–R3, although there was clearly substantial variation in reviewer scoring (Fig. S2). The proportions of whales in each focal category were as binomial random variables, and standard errors were calculated for the proportion of FOIs classified as “definite” (SE = 0.02), “probable” (SE = 0.023), “definite and probable” (SE = 0.03) and “unclassified" (SE = 0.03). Coefficients of variation (CV) derived from these estimates are shown in Table 2.

**Table 2:** Whale density estimated using satellite imagery in the Gerlache Strait, and adjusted for availability bias.

Class	Density (km^2^)	CV	Density adjusted for availability bias (km^2^)	CV
Definite	0.02	0.22	0.05	0.41
Probable	0.02	0.21	0.06	0.40
Unclassified	0.15	0.14	0.44	0.35
Definite and probable	0.04	0.14	**0.12**	0.38
Definite and probable (calmer waters)	0.04	0.15	**0.13**	0.38
Definite and probable (rougher waters)	0.02	0.47	**0.05**	0.58

Raw, unadjusted densities provided alongside combined “Definite + Probable” unadjusted densities. Densities for all classifications provided post availability bias adjustment of 0.34, along with estimated CV values. Densities provided on data from the whole image, and by image quality (i.e. rougher/calmer waters).

To account for availability bias, an adjustment of 0.34 (CV = 0.35) was applied derived from archival suction cup data collected in the same geographic area and time. Once adjusted for availability bias, and associated observer classification uncertainty, density was estimated at 0.12 whales per km^2^ (CV = 0.38). At this stage, satellite image-based densities for the whole region were lower than ship-based densities by a factor of 2.8.

**Figure 2:**
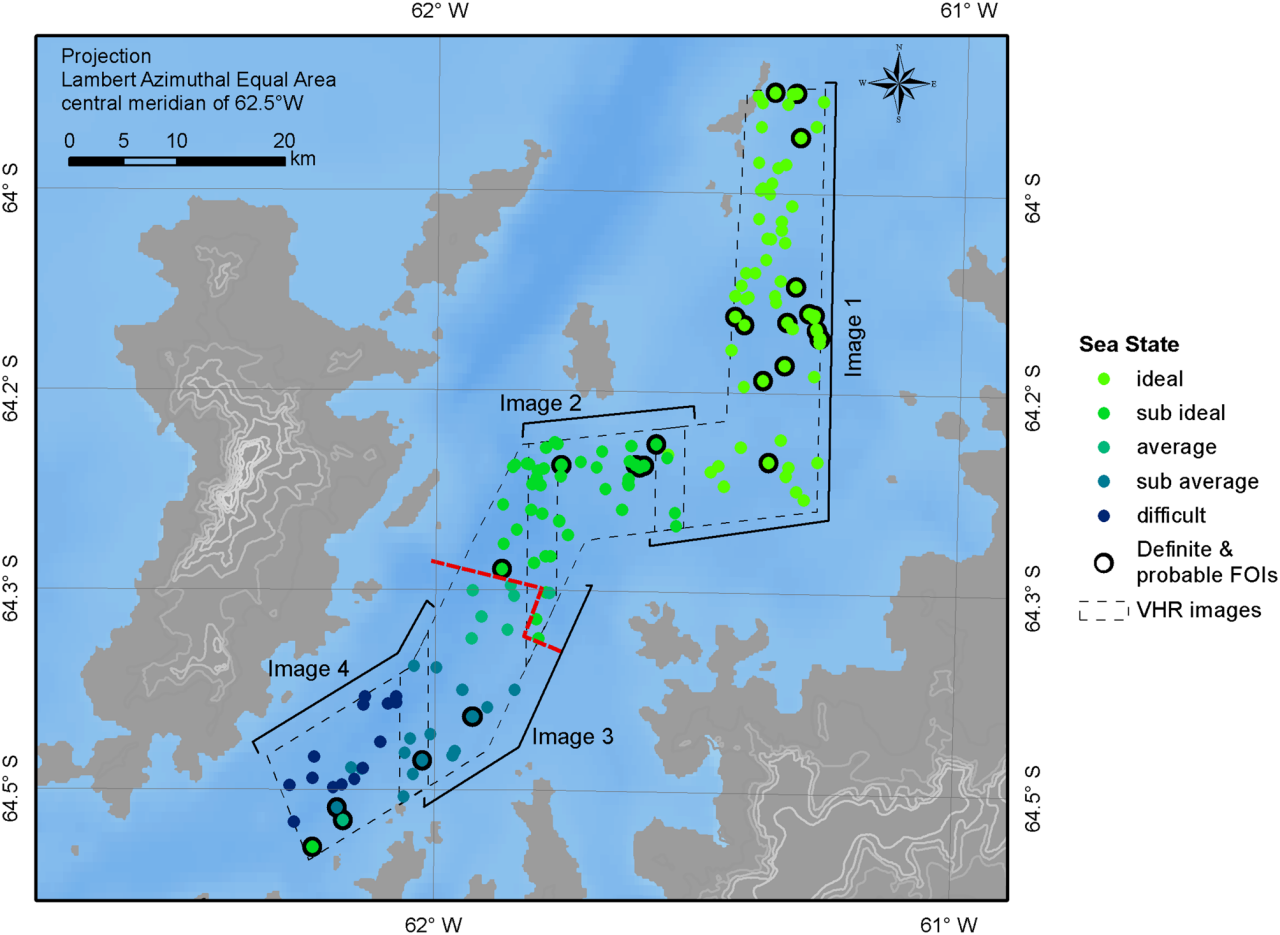
Sea state associated with each classified feature of interest (FOI) identified in the satellite imagery (coloured dots). FOIs surrounded by a black ring indicate those classified as either “definite” or “probable” whale signs. Red dotted line indicates the approximate position where the sea state transitioned from above (north of the line) to below average (south of the line). Maps were created by the authors in ESRI ArcGIS v10.6 https://www.esri.com.

Apparent sea state was recorded during the scanning and classification process. There was a marked increase in sea state from the north to the south of the study area (Fig. 2). We found that it was both easier to spot and classify FOIs in images with good to average sea states (i.e. calmer waters), which enable the FOIs to be clearly differentiated from the surface. FOIs classified as either “definite” or “probable” comprised 0.23 (SE = 0.03) for the calmer regions, versus 0.12 (SE = 0.06) for the rough water regions in the south of the study area. Were density to be calculated by grouping regions based on image quality, calmer regions (classified as good to average sea states) totalled 635 km^2^, and corresponded to a density of 0.04 whales per km^2^ (CV = 0.15); 0.13 whales per km^2^ (CV = 0.38) once adjusted for surface availability. Rougher regions (classified as sub-average and poor sea states) totalled 336 km^2^ and contained 0.02 whales per km^2^ (CV = 0.47); 0.05 whales per km^2^ (CV = 0.58) once adjusted for surface availability (Fig. 2, Table 2). Comparing between the ship and the satellite estimate from the calmer waters revealed that satellite estimates underestimated density by a factor of 2.5, and rougher regions were lower by a factor of 6.3.

## Discussion

Here we tested the capacity of VHR imagery to provide estimates useful for monitoring whale distribution and densities, using a direct comparison with a ship-based line transect survey to gauge the relative sighting rates obtained by the satellite platform in comparison to that of the ship. Our results show that density estimates derived from satellite imagery (0.13 whales per km^2^, CV = 0.38—taken from calm waters) are approximately 0.39 of those estimated from the ship-based survey (0.33 whales per km^2^, CV = 0.09); an encouraging result suggesting that data from satellite imagery has potential to detect whales at similar levels to a traditional survey method. These results match our expectation that image derived densities would be lower than that of the ship-survey, with the instantaneous nature of the image acquisition on the satellite platform likely a strong driver of these differences, in addition to limitations in image resolution and the potential for random fluctuations in local whale densities during the time between acquisition of satellite images and the vessel-based survey. However they also demonstrate that satellite surveys have sufficient whale detection capacity that they can provide a complementary approach to monitoring whale presence in remote regions where regular surveys are difficult.

In setting up this study, we chose an area that (1) is of specific scientific interest in terms of whales; (2) is remote and relatively difficult to access, but has had some whale survey effort; (3) where the environmental conditions are changing; and (4) where whale density and habitat use patterns are required to understand population recovery from exploitation and spatial overlap with the regional fishery for Antarctic krill. We focused on an area where one whale species very strongly predominates (humpback whales) in order that our results have potential use for inference about the density patterns of this species, and as there is a smaller likelihood that species mis-identification would introduce bias. We also chose a sea channel which is relatively sheltered, reducing the likelihood of turbulent sea conditions (particularly wind on sea), which can make satellite images useless for survey. Our site selection considerations highlight the limitations still facing development of VHR as a platform, and we consider these limitations and next steps to address them in the following sections. We propose that this method can be used to investigate spatial and temporal patterns of whale distribution and densities, supplementing existing methods, providing that the limitations of this new method are carefully considered during design and implementation.

Weather conditions, specifically the sea state, impact detectability of whales at sea. Sea state is known to influence the ability of observers to detect animals, with worsening conditions reducing the detection probability. Consequently, effort is typically halted when conditions exceed a predefined limit. In all at-sea surveys, sea state increases the likelihood that the assumption of perfect detection on the track line will be violated. If detection off the track line is impacted by environmental conditions, inclusion of covariates in the detection function can take account of this bias^44^ (up to a cut off, normally 5). However, if poor sighting conditions impact detection on the track line, alternative methods such as a double-observer/platform study or a mark recapture approach can be implemented to account for and quantify this bias. For an image-based survey, poorer weather conditions will also reduce the ability of the observer to differentiate FOIs from background noise (i.e. breaking waves, wind lines, etc.)^30^. This results in fewer features being identified, and lower reported densities. Poor sea state, and associated wind conditions, typically ground aerial surveys, whether manned or UAS-based, or force them to be aborted inflight. Here we show that worsening sea states in the south of the study area on the day that the image was taken (Fig. 2), correspond to lower perceived and estimated densities in these regions. Compared to the northern area, the surface conditions of the southern image were less conducive to the visual detection of FOIs, showing an increased frequency of white-caps and wind lines, possibly because this region is prone to katabatic winds sweeping into the channel. Densities in the south of the survey area, where the sea state was poorer, were 0.4 of those from calmer regions (0.05 versus 0.13 whales per km^2^, CV = 0.58 and 0.38, respectively, Table 2). To address this effect in the future, an adapted version of a Mark-Recapture Distance Sampling (MRDS) analysis, such as^45^ using multiple observers to review images^33^, could be applied to assess variations in detectability as a function of covariates (i.e. sea state), and investigate the impact of perception bias on whale detection. However, to accurately parameterise a multi-covariate model, several tens, if not hundreds of whale detections would be needed. Another approach could be to collect multiple images of the same area very close in time (within several seconds to a minute of each other), to quantify the variation in whale detections according to sea state when variation in true whale density is likely to be negligible. In the present study, density comparisons were made using data from the northern (calmer) portion of the imagery only (0.13 whales per km^2^, CV = 0.38, Table 2).

When planning satellite imagery analysis, species composition of the focal area needs to be carefully considered, because at present this approach has very limited capacity to differentiate between species when compared to in situ surveys, due to the resolution of the images (~ 30 cm in this study). Our density estimates most likely reflect the density of humpback whales using the area of the Gerlache Strait in summer, because these are the most commonly sighted species in this region, both in terms of previous surveys, where they comprise > 80% of sightings^15,16^, and during the present ship-based survey (> 95% of the groups were identified as humpback whales). During summer periods, other larger baleen whale species tend to be seen further offshore, exhibiting affinity for the more open waters of the Bransfield Strait^15^. Smaller cetacean species (e.g. Antarctic minke whales, *Balaenoptera bonarensis* and both Type A and B killer whales^46–48^, *Orcinus orca*), co-occur with humpback whales in the Gerlache Strait but are unlikely to be misidentified as humpback whales, either by ship or imagery surveys, because of their differing size, surface behaviours and morphology. Southern right whales *Eubalaena australis* are occasionally sighted in this region too^16^. However, head callosities are normally visible in overhead imagery of this species, and offer a clear means of differentiation^30,31^. Since other species likely reflect at best a very small fraction of the image-survey detections, they are unlikely to comprise a significant component of the overall density estimates.

Obtaining reliable whale density estimates require adjustments for biases. In addition to perception bias, as mentioned above, another key bias is availability bias^45^. Availability bias is the underestimation of density that occurs as a result of a proportion of animals being underwater, or too deep in the water for detection by the survey platform as it passes a point in the ocean. In the present study, we applied an estimate of surface availability^49^ (where availability is 1-availability bias), which was derived by taking dive-recording suction cup tag data from humpback whales in the same region and time, to estimate the proportion of time a whale spends at the surface, versus its dive. Applying this correction, density was initially estimated as 0.12 whales per km^2^ (CV = 0.38) over the whole region surveyed, and as 0.13 whales per km^2^ (CV = 0.38) in calmer waters. However, we note that when tag data are processed, the analyst determines the threshold at which the animal transitions from being present at the surface, to when it dives^50^. Typically, for baleen whales, dives are classified as such when the whale is > 4–5 m for > 20 s. However, with such a threshold, shallow dives of < 4–5 m would go unaccounted for. Currently, the depth to which a whale remains reliably detectable in imagery is highly variable and difficult to estimate^51^. As such, when selecting this “surface” threshold we opted for a conservative approach (< 1 m) to filter the tag data to estimate the average times a whale is visible to aerial platforms during daylight hours. We made the assumption that by applying such a shallow threshold, it would be highly unlikely that whales present above this depth would not be visible in the imagery given the resolution available and the likely turbidity of the water on the WAP. Uncertainty around the depth to which an animal remains reliably detectable is an issue for all forms of aerial surveys^26,31,52^. Additional accurate measurements of surfacing time, which include the incorporation of covariates (i.e. time of day, animal behaviour, sea state and turbidity) alongside aerial/satellite surveys, may help to more accurately account for whale surface availability in image-based surveys.

An alternative way to correct for availability bias in satellite images could be to reproduce at a “satellite-scale” the availability analyses carried out for UAV-based surveys^52,53^, whereby the surfacing rate of animals is captured in video or multiple overlapping still images, and availability estimated. Logistically, repeating this with satellite imagery may be more challenging, given orbital acquisition windows, but the possibility exists to examine surface availability in overlapping sequential images. However, surface availability is a highly variable process^52^. Thus, to correct for it requires careful consideration on a case-by-case basis, and using adjustments stemming from data collected in spatially and temporally comparable regions. Whilst possible for this study, we note that it is not realistic to assume that estimates of surface availability will be available for all regions. However, we would recommend that steps are taken to obtain such estimates, and for future image-based surveys to apply corrections derived from data in close proximity, both spatially and temporally, to the focal region.

The surface availability adjustments made in the current study are akin to those typically made for a ship or manned aerial surveys to account for diving behaviour of the target species^45^. One component of the issues of availability that these adjustments cannot cover, however, is the speed of satellite image acquisition. Satellites survey vast areas in seconds, a process which exaggerates the effect of availability bias, and, therefore, decreases the number of animals which can be detected in comparison to ship or aerial surveys. Further investigation of the relationship between instantaneous surface availability and image properties, perhaps through using images repeatedly collected over short time periods, as mentioned above, in conjunction with other local surveys via ship, UAS or “circle back” methods to simultaneously estimate visibility bias (i.e. a combination of both availability and perception bias)^54^.

Perception bias potentially has differing effects in a ship-survey versus an image survey. In a typical line or point transect survey, perception bias is introduced when an animal, which is available for detection, is missed by the observers for whatever reason^45^, these include, but are not limited to, observer fatigue, worsening environmental conditions, observer inexperience, and chance. However, in an image-based survey, perception bias manifests itself slightly differently, given the extended period available to observers to review the images of the surface of the ocean. This longer review time, and the ability to rest observers, without losing in situ survey time, probably reduces the likelihood of an observer missing an animal if it was there, at the surface, to be detected—but further research is needed to test that assumption. In this instance, perception bias is reflected in variations in how FOIs are classified. Here we compared between the scores given for a randomly chosen subset of the original data, and the re-classified scores from three independent reviewers. We found that despite a degree of inter-user variation (Fig S2) the variance in the scores did not exceed 1 (Fig S1), and when averaged over the three reviewers, the proportions of these scores classified as either “definite” or “probable”, did not deviate significantly from the original data. This suggests that at an FOI-level, inter-user variation was present, presumably reflecting an individual’s interpretation of what the feature being considered was. However, averaging over this variability in individual perception still revealed a very similar proportion of FOIs that were classified and scored as “definite” and “probable” whales compared to the main observer. Despite this, we note that variation between observers represents a sizable source of uncertainty associated with manual scanning and classification of imagery, with data being prone to user performance bias. Automation of the initial image scanning and FOI classification process using machine learning tools could go some way to solving this issue. Automation would not totally remove perception bias, as the parameters offered to define/train the automated systems would themselves be subject to a degree of bias. However, trained algorithms with known, quantifiable uncertainties may provide a more analytically uniform means of scanning, identifying and classifying FOIs. Studies^55,56^ have shown remarkable progress in this field over recent years,however ongoing development and testing is still required to hone these methods in order to assess their accuracy when compared to visual observations in challenging conditions.

Further classifications using larger numbers of human observers (e.g. crowd sourced analysis of imagery^57^ could also provide a useful means of optimising the approach, to: (1) provide a best practice approach to classification which reduces inconsistent interpretation among observers, and (2) provide overall better perception of FOIs averaged over a larger number of observers, reducing error brought about by extreme differences in individual perception. Currently, there is not enough data for both approaches to adequately parameterise the observation process, both require substantially larger data sets of whales in satellite imagery than are currently available, and as such this remains an area of interest for future research.

Satellite imagery as a platform for assessing whale occupancy is in its infancy but this assessment shows that with careful consideration of location and environmental conditions, it can provide density estimates which could be useful for monitoring whale density patterns in time and space for some populations, complementing existing methodologies. There are a number of key areas in which image-based surveys need to be developed to ascertain their overall comparability to existing techniques, for example via continued data collection, careful consideration of environmental conditions, and further assessment of instantaneous surface availability. However, one area where satellite imagery is distinctly advantageous, is that it has potential to survey very large areas instantaneously, providing weather conditions can be accounted for. This allows for more simple analysis than traditional line transect, as the latter requires extrapolation between transects in order to infer broader-scale density estimates. Reliable species identification would also represent a significant milestone in the development of this method. The results presented here act as a first attempt, and a baseline from which future studies can focus on addressing the aforementioned limitations. Global ecosystems are moving through a period of increased perturbation^23,58,59^, where costs and limited access are hampering research. The ability to deploy satellites to collect data offers a distinct advantage over existing survey techniques, which are expensive, use high volumes of fuel and often face significant logistical lead times compared to the effective “real-time” assessments that can be made through remote earth observations. Our results show that VHR satellite imagery has strong potential to be used as a safer, non-invasive means of surveying remote regions, which compliments existing approaches.

## Methods

**Figure 3:**
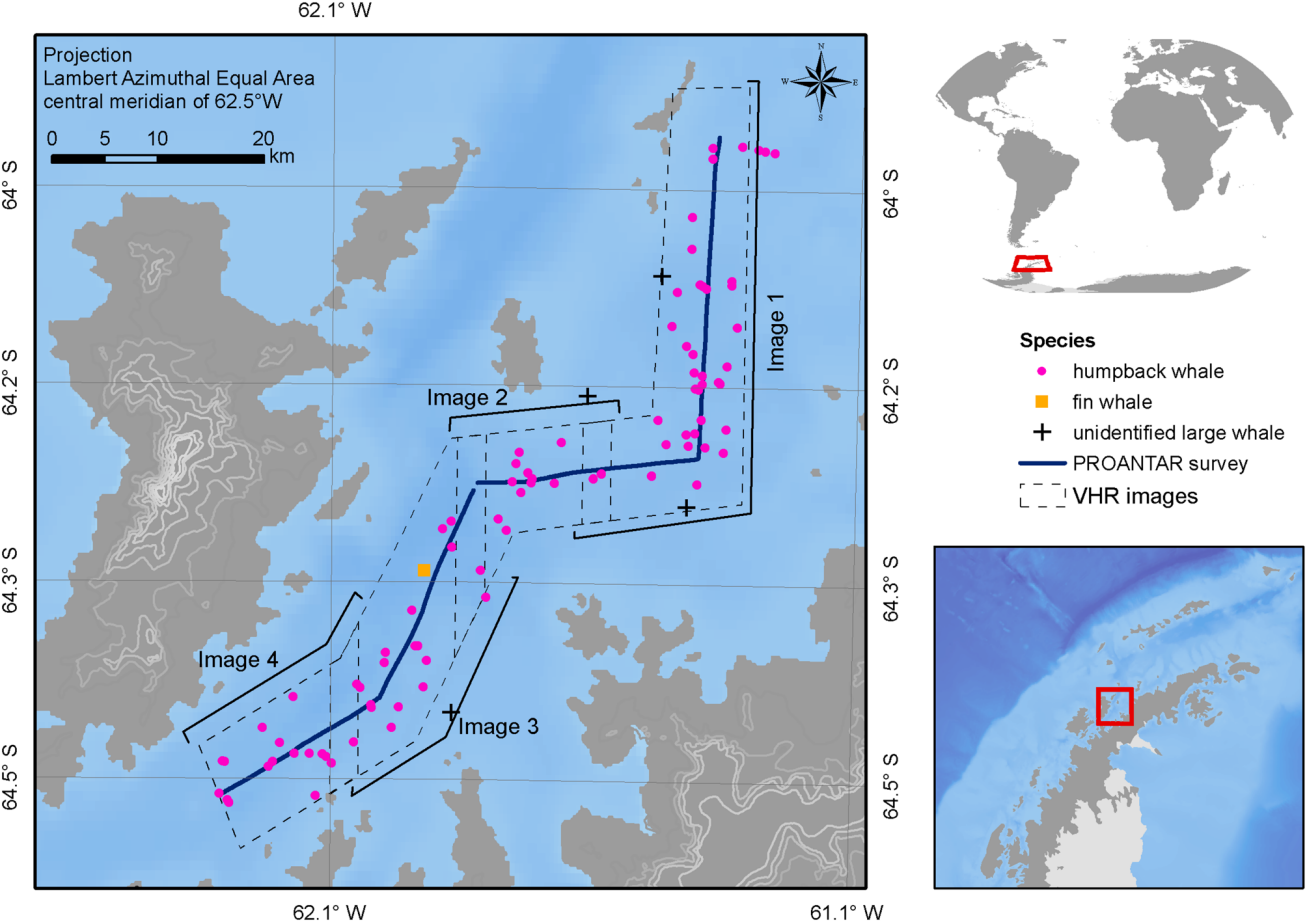
Gerlache Strait, Western Antarctic Peninsula depicting the on-effort ship-survey transects (blue line), sightings (pink dots, humpback whale, *Megaptera novaeangliae*; black cross, unidentified large whale; orange square, fin whale, *Balaenoptera physalus*). Footprints of the acquired WorldView-3 images are depicted by dashed-boxes. Maps were created by the authors in ESRI ArcGIS v10.6 https://www.esri.com.

Satellite imagery was purchased to coordinate with a section of the whale sighting surveys conducted by the Brazilian Antarctic Program (PROANTAR), who surveyed the Gerlache Strait on 25 February 2018 (Fig. 3). This survey was timed to coordinate with peak whale occurrence on the WAP, estimated to be from late summer into autumn^60^.

### Ship-based survey protocol

Line-transect procedures operating under passing mode (where the vessel did not close to confirm sightings or group size) were implemented on the 93 m Polar vessel NPo “Almirante Maximiano”, of the Brazilian Navy. Radial distances and bearings (relative to the heading) using 7 × 50 reticule binoculars and angleboards, respectively, were recorded to obtain perpendicular distances to the sightings from the trackline^1,61^. Two observers were stationed at 14.6 m above sea level, with one scanning port and the other starboard forward of the beam. Effort was focused towards the transect line with an overlap of about 10° on the bow. Data were collected with the ship moving at 10 knots and in sea states below Beaufort 5. To minimise fatigue, the five observers were rotated every 30 min (two on effort, one data recorder and two resting) and environmental conditions were recorded at this rotation, or when conditions changed. Effort was halted in sea states above Beaufort 5 or when visibility dropped below 3 nautical miles. Care was taken to avoid the introduction of duplicate sightings within the 10° overlap at the bow. All data including bearings, reticule measurements, species, group size and composition were recorded using *Logger* 2010^62^, and observers were instructed to be accurate with reticule and angle measurements.

Geographic constraints of the Gerlache Strait, combined with passage regulations, meant that the survey was unable to implement a design allowing for an equal probability of coverage, for example to extrapolate density estimates beyond sampled regions. The PROANTAR surveys were designed to enable repeated measurement of a highly ice-dynamic region, where passage is often limited to the channel, thus they are simplified to enable estimates of relative abundance over time to be made within the footprint covered. Therefore, density estimations presented herein are applicable only to the area covered by the transects and are not extrapolated to provide regional estimates. Only on-effort sightings were included and densities provided here are defined per square kilometre (km^2^). Distance sampling surveys are based on an assumption that all animals present on the transect line are sighted, i.e., $g\left( 0 \right) = 1$, where $g\left( x \right)$ is the probability of detecting an animal at distance $x$. For cetaceans, the detection probability, $g\left( x \right)$, is not assumed to be 1^63^, as a consequence of their diving behaviours, and needs to be accounted for if estimates are to be accurate. The detection probability is influenced by both species-specific surface availability bias, and observer mediated perception bias, where animals that are available to be detected are missed by the observers^45^. The effect of surface availability on $g\left( 0 \right)$ can be accounted for by providing an upward correction to estimates, whilst perception bias can be minimised through the use of experienced observers, training, and rotation to mitigate fatigue. No adjustment for $g\left( x \right)$ were made to the ship survey data, as given the highly conspicuous surface behaviour of humpback whales (> 95% of sightings) it is presumed unlikely that the observers would have missed sighting a whale present on the transect line, particularly given the observer bow-overlap. Furthermore, Johnston et al.^16^ demonstrate, using tag data, that for humpbacks in Gerlache Strait, $g\left( 0 \right) \approx 1$. In the absence of associated tagging data for the present study, we assume this is also the case for the ship-based survey conducted here. Sightings data were analysed in the “Distance” package^64^ in R v3.5.5^65^. A multiple covariate distance sampling (MCDS) framework was applied to groups of animals, assuming $g\left( 0 \right) = 1$. Covariates tested included Beaufort sea state, visibility, and sightability. Visibility was measured on a linear scale described by: Good (horizon clearly visible); Fair (no horizon, but visibility > 3 nautical miles); Poor (visibility < 3 nautical miles). Sightability was defined as: 0 (excellent); 1 (good); 2 (moderate); 3 (poor) based on conditions required to reliably spot a minke whale. Perpendicular distance truncation at 5% was tested. Half-normal and hazard rate keys were tested using no adjustment terms to fit the detection function. Model selection was based on minimum Akaike’s information criterion (AIC) values^66^, and checking of the parameter estimates.

### VHR image collection and analysis

**Figure 4:**
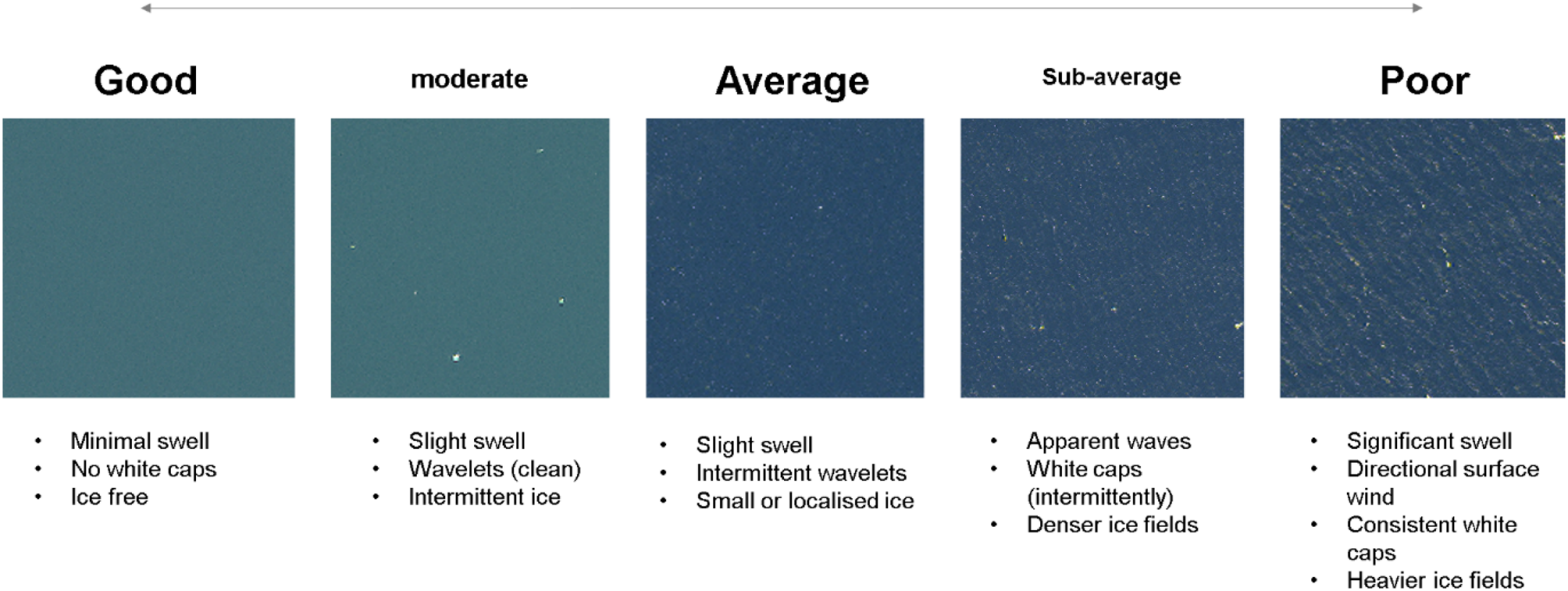
Linear scale of sea state showing that as sea state increases, the overall image quality decreases, increasing the time required and the difficulty of the scanning process. Satellite image ©2020 Maxar Technologies.

Four WorldView-3 images were acquired totalling 866 km^2^ (971 km^2^, including overlaps). Images 1 to 3 were taken on the 15 February 2018 and image 4 on 17 February 2018 (Fig. 3). Care was taken during analysis to prevent the introduction of double counts of features from overlapping images, and images were scanned twice to prevent any FOIs from being erroneously omitted. Given the ability to examine the images for extended periods, taking breaks regularly, and revisiting the entire region twice, the likelihood of missing FOIs, if present in the image, was assumed to be nil. Animal movement into/out of the study area was assumed to be no more likely than that encountered during a multi-day vessel survey, and therefore double-counting was presumed not to influence the results. Images were scanned systematically by eye using a 0.5 km by 0.5 km grid at a scale of 1:2,300 by experienced observers. Apparent sea state around the FOI was also recorded during the imagery classification process using a linear scale between “ideal” and “poor” indicating an increasing sea state (Fig. 4).

Image analysis followed that described in Cubaynes et al.^31^. Images were first loaded into ArcGIS v.10.4 (ESRI, 2017), and pan-sharpened using the ESRI algorithm,a process in which a higher resolution panchromatic image (0.31 m) is used to enhance a coarser resolution multispectral image (1.24 m), yielding a high resolution multispectral composite. In order to assess each whale-like feature in the image, candidate FOIs were identified and classified using 13 distinct criteria taken from Cubaynes et al.^31^. These criteria are indicative of whale-like characteristics that (1) stand out from the homogeneous environment, (2) are not easily replicated by background conditions, and (3) closely align with the whale identification criteria implemented by ship-surveys. Criteria were scored between 0 and 2, with 0 indicating that the criterion was not met, and the FOI did not conform to a whale-like feature,scored as 1 if the FOI indicated partial conformity, typically characterised by blurred edges and reduced clarity overall, and 2 if the FOI conformed to the criterion and thus indicated a whale-like feature. Scores for each criteria ($C_{s}$) were combined following:

1 $$C_{s} = \sum \left( {\left( {\psi_{1} + \psi_{2} + \psi_{3} + \psi_{4} \left( { \cdot 2} \right)} \right) + \psi_{5} + \cdots \psi_{13} } \right)$$

Weighting factors were assigned to Ψ_1_ through Ψ_3,_ corresponding to visible fluke, fins and footprints in line with Cubaynes et al.^31^. In this study we also weighted blow signs, Ψ_4_, by a factor of 2 in the effort to improve inter-platform comparability, as these criteria are highly indicative of a whale and are commonly used when sighting from a ship (i.e. if a visible blow is spotted, the sighting would be confirmed and recorded, and the weighting factor is designed to reflect this in the image survey). $C_{s}$ for each whale was classified as either “definite”, “probable” or “unclassified” FOIs based on the scoring system, where $C_{s}$ > 9, "definite", if ≥ 7, "probable", and < 7, "unclassified”.

The initial scanning and classification of the imagery and FOIs was carried out by a single observer (O1). To investigate the effect of inter-user variation, a randomly chosen subset of FOIs (n = 37, 20%) were reclassified by three independent reviewers (R1-R3). We note, that as with at-sea marine mammal sightings, a level of previous experience is likely to improve observations, and as such, experienced cetacean observers were used to review the images. The average classification from these observers were then compared to those of the O1, to assess the pattern of deviance between scores. If these were significantly different from O1, an adjustment to the original FOIs scores was anticipated. The proportion of the subset of the FOIs identified as either “definite” or “probable” was also estimated for O1, R1, R2 and R3.

Whale densities were estimated using the counts of “definite” and “probable” FOIs. These two criteria represent identified features that resemble and are highly likely to be a whale. “Unclassified” FOIs typically represent unusual surface disturbances, which can be scored according to some visibility criteria (e.g. the disturbances are of a similar length, shape or width of a whale), but are not visually indicative of a whale-like feature when examined in detail. The total set of scored FOIs from O1 were fitted to a negative binomial distribution (Fig S3) using the R package “fitdistplus”^67^. To obtain a measure of the classification uncertainty we assumed a binomial model, and used this to calculate the standard error of the proportion ($p$) to total FOIs ($n$) classified as either “definite”, “probable” or “unclassified”, where standard error was calculated as $\sqrt {p\left( {1 - p} \right)/n}$.

Since satellite imagery acquisition is instantaneous, the combined total of “definite” and “probable” FOIs were then corrected for availability bias. Availability bias was estimated based on an instantaneous viewing time. In order estimate surface availability, suction cup archival tags with video and 3D accelerometery data from customised Animal Tracking Solutions (https://www.CATS.is, as described in Cade et al.^68^, were deployed on 21 whales in February 2017, 2018 and 2019 in bays surrounding the study area, remaining attached and recording data for an average of 20.08 ± 0.7 h. Depth data (collected at 10 Hz) was used to determine the mean daylight surface time, $E_{s}$ and the mean dive time of each animal, $E_{d}$ (Table 3). Using these data, $E_{s}$, and $E_{d}$ were calculated from each of the individual whale IDs reported. Availability, $\hat{a}$, weighted by tag duration, was estimated to be 0.34 (CV = 0.35), and density estimate, $\hat{d}$, was then corrected for availability bias, $\hat{\alpha }$, by $\hat{d}/\hat{\alpha }$.

**Table 3:** Surface and dive times to the nearest second (s) of humpback whales (*Megaptera novaeangliae*) tagged with digital time-depth devices in the Gerlache and surrounding bays during February 2017, 2018, and 2019.

Deployment ID	Time above 1 m (s)	Time below 1 m (s)	Total daylight duration of tag (s)	Proportion of time at < 1 m
mn170218-31	16,186.2	20,824.0	37,010.2	0.44
mn170220-30	3,290.3	5,561.6	8,851.9	0.37
mn180227-40	11,330.8	12,016.0	23,346.8	0.49
mn180227-41	23,012.5	37,736.1	60,748.6	0.38
mn180227-43	27,609.2	36,505.8	64,115.0	0.43
mn180227-44	25,090.5	69,660.9	94,751.4	0.26
mn180227-45	212.4	685.2	897.6	0.24
mn180227-46	17,653.9	46,775.4	64,429.3	0.27
mn180227-47	309.8	619.5	929.3	0.33
mn180228-47	12,358.4	29,965.0	42,323.4	0.29
mn190203-22	24,306.4	71,692.5	95,998.9	0.25
mn190203-23	12,394.2	25,526.3	37,920.5	0.33
mn190205-27	19,229.8	45,189.7	64,419.5	0.30
mn190205-40	23,349.2	63,803.5	87,152.7	0.27
mn190212-27	6,658.6	18,529.3	25,187.9	0.26
mn190212-40	3,146.4	18,547.3	21,693.7	0.15
mn190215-40	7,653.3	35,539.3	43,192.6	0.18
mn190225-40	12,137.1	15,920.2	28,057.3	0.43
mn190225-44	1,448.0	992.8	2,440.8	0.59
mn190228-42	41,582.5	51,995.0	93,577.5	0.44
mn190228-44	27,466.5	11,853.8	39,320.3	0.70
Mean	15,068	29,521	44,589	0.34
SE	2,403	4,869	6,802	0.03

Mean of the proportion of time at < 1 m depth provided is weighted by tag duration.

## Supplementary information

Supplementary file 1

## Data Availability

WorldView-3 images analysed herein were licensed from DigitalGlobe Inc., a subsidiary of Maxar Technologies Inc., and are available to purchase from the archive at https://discover.digitalglobe.com/, using image ID numbers: 1040010038AFA900, 1040010039052600, 104001003757E500, and 104001003AC95B00. Ship survey data is being held under an embargo period by the Brazilian Antarctic Program., please contact LDR. Bathymetry data visualised is available freely from GEBCO_2014 30-arc second grid, version 20150318, https://www.gebco.net.
