# Supplementary material for: A comparison of baleen whale density estimates derived from overlapping satellite imagery and a shipborne survey
Source: Sci Rep. 2020 Jul 31;10:12985. doi: 10.1038/s41598-020-69887-y (PMC7395155; doi:10.1038/s41598-020-69887-y)
Supplement: Supplementary file 1 [file 41598_2020_69887_MOESM1_ESM.pdf]

A comparison of baleen whale density estimates derived from overlapping satellite imagery and a shipborne survey

[*1,2]Bamford, C.C.G.,[3]Kelly, N., [4]Dalla Rosa, L., [5,6]Cade, D.E. [1]Fretwell, P., [1]Trathan, P.N., [1]Cubaynes, H., [4]Mesquita, A., [1]Gerrish, L., [5]Friedlaender, A.S., and [1]Jackson, J.A.

1. British Antarctic Survey, High Cross, Madingley Road, CB3 0ET, Cambridge, United Kingdom

2. University of Southampton, University Road, Southampton, SO17 1BJ, United Kingdom

3. Australian Antarctic Division, Department of Agriculture, Water and the Environment, Australian Government, Channel Highway, Kingston 7050, Australia

4. Laboratório de Ecologia e Conservação da Megafauna Marinha, Instituto de Oceanografia, Universidade Federal do Rio Grande – FURG, Av. Itália km.8, Rio Grande – RS 96203-900, Brazil

5. Hopkins Marine Station, Stanford University, 120 Ocean View Blvd, Pacific Grove, CA 93950, USA.

6. Institute for Marine Sciences, University of California Santa Cruise, 115 McAllister Way, Santa Cruz, CA 95006, USA

***corresponding author: conord48@bas.ac.uk**

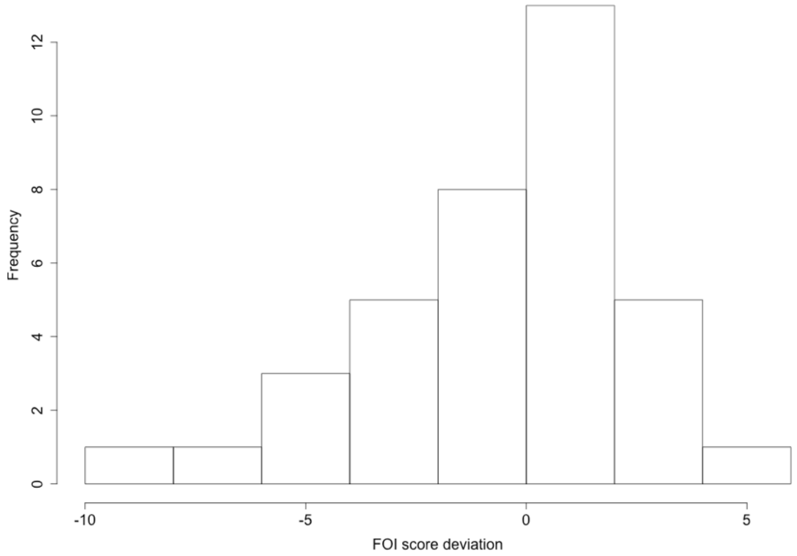

Fig S 1 – Histogram of the scores of the features of interest (FOIs) from the three independent reviewers illustrating the shift in scorings, which were either left (lower than) or right (higher than) the original FOI score, which here can be interpreted as a deviation of 0.

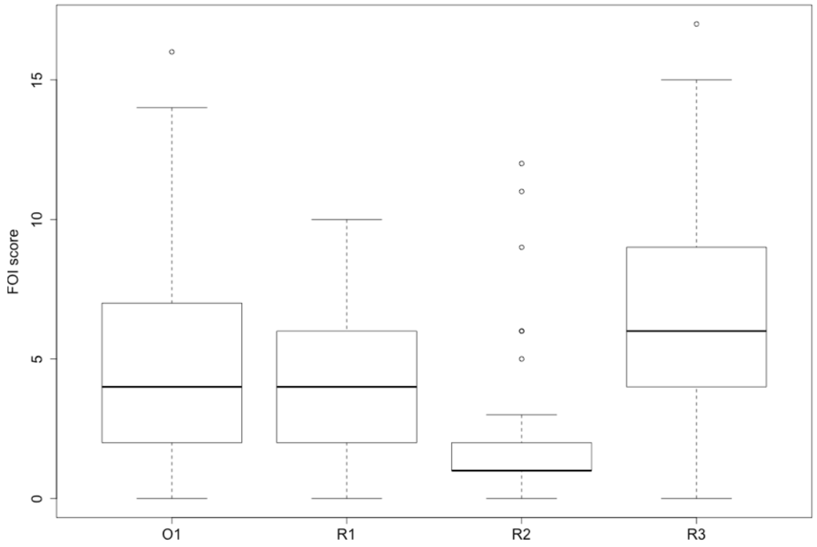

Fig S 2 – Scores of the features of interests (FOIs) from the review subset (n=37) for the main observer (O1) and the three reviewers (R1 to R3).

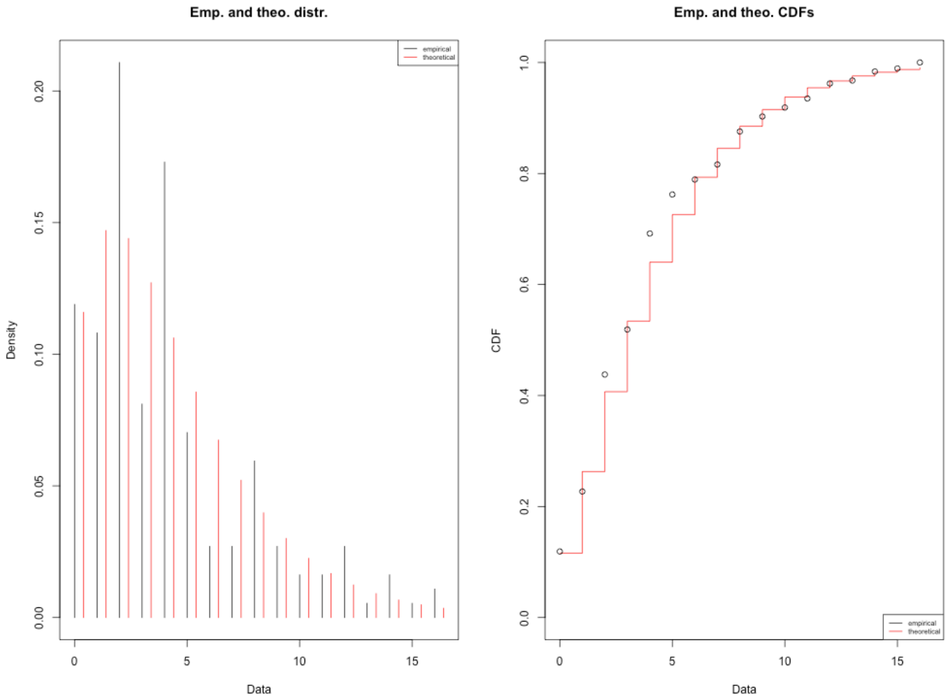

Fig S 3 – Fit of the scores of the classified features of interest (black) to a theoretical negative binomial (red) produced in the R package "fitdistrplus" [1]*.*

References

1 Delignette-Muller, M. L. & Dutang, C. fitdistrplus: An R package for fitting distributions. *Journal of statistical software* **64**, 1-34 (2015).
